# Wet and Dry Atmospheric Depositions of Inorganic Nitrogen during Plant Growing Season in the Coastal Zone of Yellow River Delta

**DOI:** 10.1155/2014/949213

**Published:** 2014-04-01

**Authors:** Junbao Yu, Kai Ning, Yunzhao Li, Siyao Du, Guangxuan Han, Qinghui Xing, Huifeng Wu, Guangmei Wang, Yongjun Gao

**Affiliations:** ^1^Key Laboratory of Coastal Zone Environmental Processes and Ecological Remediation, Yantai Institute of Coastal Zone Research (YIC), Chinese Academy of Sciences (CAS) and Shandong Provincial Key Laboratory of Coastal Zone Environmental Processes, YICCAS, Yantai 264003, China; ^2^University of Chinese Academy of Sciences, Beijing 100049, China; ^3^College of Environmental Science and Engineer, Ocean University of China, China; ^4^Department of Geosciences, University of Houston, Houston, TX 77204, USA

## Abstract

The ecological problems caused by dry and wet deposition of atmospheric nitrogen have been widespread concern in the world. In this study, wet and dry atmospheric depositions were monitored in plant growing season in the coastal zone of the Yellow River Delta (YRD) using automatic sampling equipment. The results showed that SO_4_^2−^ and Na^+^ were the predominant anion and cation, respectively, in both wet and dry atmospheric depositions. The total atmospheric nitrogen deposition was ~2264.24 mg m^−2^, in which dry atmospheric nitrogen deposition was about 32.02%. The highest values of dry and wet atmospheric nitrogen deposition appeared in May and August, respectively. In the studied area, NO_3_^−^–N was the main nitrogen form in dry deposition, while the predominant nitrogen in wet atmospheric deposition was NH_4_^+^–N with ~56.51% of total wet atmospheric nitrogen deposition. The average monthly attribution rate of atmospheric deposition of NO_3_^−^–N and NH_4_^+^–N was ~31.38% and ~20.50% for the contents of NO_3_^−^–N and NH_4_^+^–N in 0–10 cm soil layer, respectively, suggested that the atmospheric nitrogen was one of main sources for soil nitrogen in coastal zone of the YRD.

## 1. Introduction

It is well known that nitrogen is an important nutrient in terrestrial and marine ecosystems. The primary production, the nutrient cycling, and the biodiversity in natural ecosystems were great limited by the availability of reactive nitrogen [1–5]. The global reactive nitrogen production rate increased from approximately 15 Tg N yr^−1^ in 1860 to 187 Tg N yr^−1^ in 2005; more than half of total was deposited onto the ground [6]. Atmospheric nitrogen deposition has become a large source of nitrogen for terrestrial and aquatic ecosystems worldwide [7]. The atmospheric nitrogen deposition can affect the soil nitrogen balance, which probably results in some negative effects on terrestrial and marine ecosystems [8–10] through eutrophication and acidification [11]. Nitrogen entering the soil-plant system has been a main factor for the nitrogen cycle of ecosystem [12]. Atmospheric nitrogen deposition has frequently been observed to increase soil carbon (C) storage in natural ecosystems [13, 14]. Some studies tried to build relations between atmospheric depositions and nitrogen concentration in moss and proved that mosses could serve as biological indicators for atmospheric nitrogen depositions [15]. Therefore, the atmospheric nitrogen deposition has become an increasingly important source for reactive nitrogen entering to the coastal ecosystems and contributed to the coastal nitrogen budget [5].

The ecological problems caused by dry and wet deposition of atmospheric nitrogen have widespread concern in the world. In recent decades, high rates of atmospheric nitrogen deposition have been reported in Europe [16], East Asia [17], North America [18], and Northern and Southeastern China [19, 20]. The National Atmospheric Deposition Program (NADP) was built to oversee the long-term sampling and analysis of precipitation across the United States, Puerto Rico, and the Virgin Islands [21], and the dry deposition was monitored by networks in Europe (EMEP), North America (CASTNET), and East Asia (EANET). As the largest developing country, China has consumed more than 24 Tg year^−1^ fertilizer N in recent years, which is ~30% of total fertilizer N used worldwide [22]. In addition, livestock production in China has increased greatly since the late 1980s. The amount of NH_3_ volatilization from wastes of domestic animals (manure and urine) is even higher than that from fertilizer use in China [23]. Furthermore, the transport network and traffic have increased rapidly since the 1980s in China, resulting in increasing NO_*x*_ emissions by 62% [24]. 70–80% of the emitted nitrogen was deposited to the land or water surface as wet and dry deposition [11]. Nitrogen deposition was the highest over Southern China and exhibited a decreasing gradient from Southern to Western and Northern China. The anthropogenic activities were the main reason for the nitrogen deposition increase [25]. Therefore, China is now a hotspot for nitrogen deposition according to recent modeling studies [7, 25, 26]. However, the magnitude of atmospheric deposition of various N species in China remains uncertain because of a paucity of measurements and quantitative knowledge [19, 27]. Previous studies of atmospheric nitrogen deposition in China have considered the wet and the dry deposition separately [28, 29] and most monitored locations were in agricultural areas and cities [19, 22]. Few measurements have focused on both the wet and the dry deposition of individual nitrogen species in coastal wetlands. In this study, the dry and wet nitrogen deposition was monitored using automatic sampling equipment in coastal wetland of the Yellow River Delta (YRD) which is one of intensive agricultural regions and rapidly economic developing regions in China. The objectives of the present study were to (1) determine the composition and amount in dry and wet atmospheric nitrogen deposition, (2) reveal the monthly variation of nitrogen (wet/dry) deposition in growing season, and (3) assess the contribution of atmospheric nitrogen inputs to local soil.

## 2. Materials and Methods

### 2.1. Study Area Description

**Figure 1:**
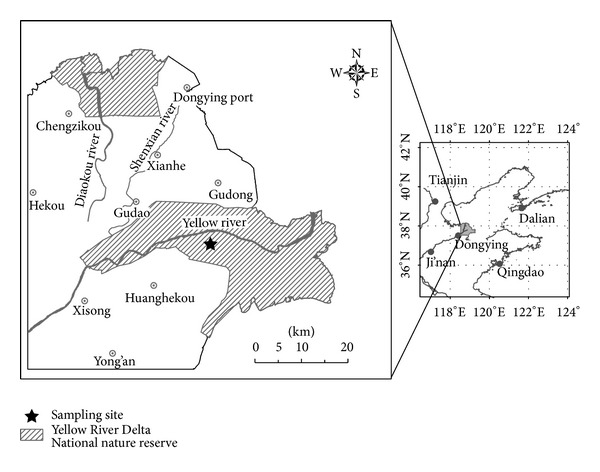
The location of the Yellow River Delta and sampling sites.

The sampling sites (118°58′E, 37°45′N) located at the Yellow River Delta Ecology Research Station of Coastal Wetland, Chinese Academy of Sciences (Figure 1), which is in the Yellow River DeltaNational Nature Reserve. All around the sampling sites was wide open without buildings. The climate in study area is warm temperate continental monsoon climate. It is arid and windy in spring, hot and rainy in summer, cool and sunny in autumn, and less snowy and dry in winter [30]. The annual average temperature is ~12.1°C, the annual average precipitation is ~551.6 mm, and the annual average evaporation is ~1962 mm. More than 85% of plants are the aquatic vegetation and halophytic vegetation in study region. The* Suaeda salsa* and* Phragmites communis Trin* are predominant plants and widely distributed [27].

### 2.2. Sampling

The dry and wet atmospheric depositions were monitored in plant growing season from May to November in 2012. The samples were collected using SCJ-302 model automatic sampling equipment (Qingdao Xuanhui Instruments & Equipment Co. Ltd, China). The sensitivity of the equipment was 0.05 mm/h. The automatic sampling equipment stops the dry atmospheric deposition collection with a lid covered and starts to collect the wet atmospheric depositions sample within 60 seconds of rainfall event beginning. As soon as the precipitation ceased, the head covering covered over the wet atmospheric deposition collection buckets and rotated to collect the dry atmospheric deposition. Meanwhile, the TE525 tipping bucket gauge (Texas Electronics, USA) which was anchored 0.7 m above the ground was used to monitor precipitation. In this study, the method of Balestrini et al. [31] was used for sample collection. According to national atmospheric environmental monitoring criterions, the solution of ethylene glycol was used at the surface of collection bucket to collect the dry atmospheric deposition samples.

The dry atmospheric deposition samples were collected monthly and wet atmospheric deposition samples were collected after each precipitation event. In the monitoring period, the surface soils (0–10 cm) in atmospheric deposition monitoring sites were collected monthly.

### 2.3. Analytical Procedures

The wet and dry deposition samples were taken to the laboratory for chemical analysis. The water-soluble ions (Na^+^, K^+^, Ca^2+^, Mg^2+^, NH_4_^+^, Cl^−^, NO_3_^−^, and SO_4_^2−^) were measured by ICS3000 ion chromatograph (Dionex, USA). Total inorganic nitrogen (TIN) was considered as the sum of ammonium nitrogen (NH_4_^+^–N) and nitrate nitrogen (NO_3_^−^–N). The number of ions and nitrogen content per unit area for wet and dry deposition samples was calculated using cross-sectional area and volume of wet and dry atmospheric deposition collection buckets.

The air-dried soil samples which collected in monitoring sites were extracted in 2 mol/L KCl. Then the contents of NH_4_^+^–N and NO_3_^−^–N were analyzed by a flow-injection autoanalyzer (Seal-Branlubbe AA3, Seal Germany). The soil volume weight was measured by cutting ring method.

### 2.4. Statistic Analyses

The data were statistically analyzed by the descriptive statistics and personal correlation coefficient. The significance was defined if the probability value (*P*) of a test is less than 0.05.

## 3. Results and Discussion

### 3.1. Results

#### 3.1.1. Ionic Composition and Ion Concentrations in Atmospheric Deposition

**Figure 2:**
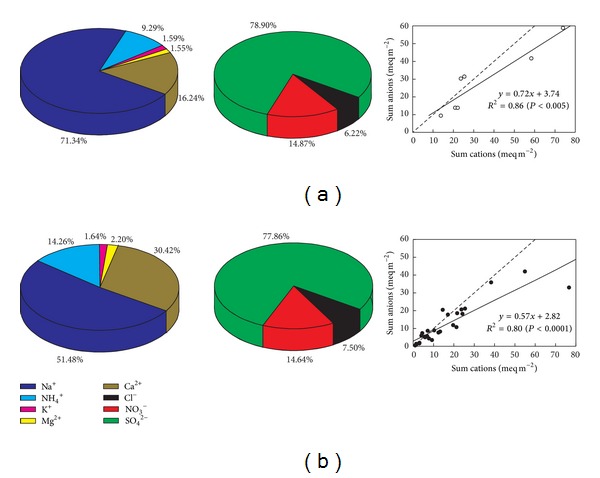
The percentage of water-soluble ions and the ionic balance in dry (a) and wet (b) atmospheric deposition.

The major cations and anions of ionic compositions were Na^+^ and SO_4_^2−^ in dry and wet atmospheric deposition in the YRD, respectively (Figure 2). The predominant cation in dry atmospheric deposition was Na^+^ (71.34%), followed by Ca^2+^ (16.24%) and NH_4_^+^ (9.29%). These three cations accounted for more than 95% of the total cations in dry atmospheric deposition, while the total number of K^+^ and Mg^2+^ was less than 5%. The major anions in dry atmospheric deposition were SO_4_^2−^ and NO_3_^−^, which were more than 93% of the number of the total anions (Figure 2(a)). Ionic composition in wet atmospheric deposition was similar to that in dry deposition. Compared to the dry atmospheric deposition, the proportions of Ca^2+^ (30.42%) and NH_4_^+^ (14.26%) in wet atmospheric deposition were relative high. SO_4_^2−^ constituted ~77.86% of the total cation numbers and was also the predominant anion in wet atmospheric deposition (Figure 2(b)).

The significant relations of the total number of anions and the total number of cations were observed in both dry atmospheric depositions (*P* < 0.005) and wet atmospheric depositions (*P* < 0.0001) (Figure 2). The correlation coefficients (*R* ^2^) were 0.86 and 0.80, respectively.

#### 3.1.2. Monthly Variations of Atmospheric Nitrogen Depositions

**Figure 3:**
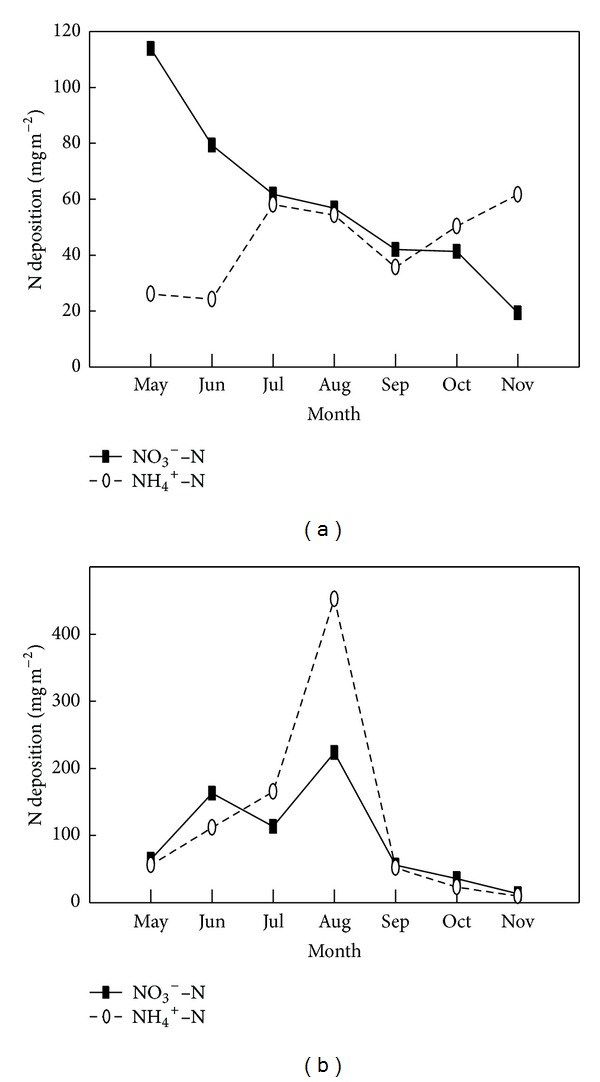
The monthly variations of NO_3_^−^–N and NH_4_^+^–N in dry (a) and wet (b) atmospheric depositions.

The main type of nitrogen in dry deposition was NO_3_^−^–N (~57.21%). The maximum values of TIN and NO_3_^−^–N in dry atmospheric deposition were 139.99 mg m^−2^ and 113.89 mg m^−2^, respectively, which was observed in May (Table 1 and Figure 3(a)). The main nitrogen in wet deposition was NH_4_^+^–N which accounted for ~56.51%. The high content of NH_4_^+^–N in wet deposition was observed from June to August when the rainfall was abundant (Figure 3(b)). There was a significant positive relationship between the content of NH_4_^+^–N in wet deposition and precipitation in the study (*R* ^2^ = 0.90). In addition, the fertilizer was widely used in this period. High temperature accelerated ammonia volatilization in wetland ecosystem and large quantity ammonia application caused the content of NH_4_^+^–N to increase. Therefore the peaks of precipitation (~297.3 mm) and the content of NH_4_^+^–N (452.24 mg m^−2^) in wet deposition occurred simultaneously in August (Figure 3(b)). However the content of NO_3_^−^–N in wet deposition varied with precipitation was not obvious. During the study period, the contributions of NO_3_^−^–N and that of NH_4_^+^–N to total atmospheric deposition were ~48% and ~52%, respectively (Table 1).

**Figure 4:**
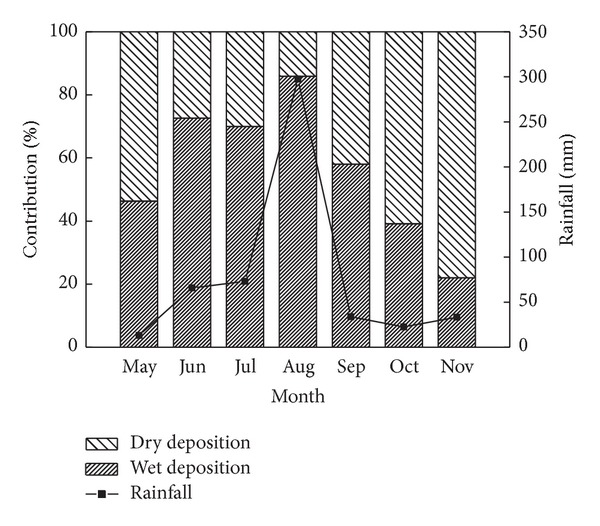
The variation of contributions of wet and dry deposition.

**Figure 5:**
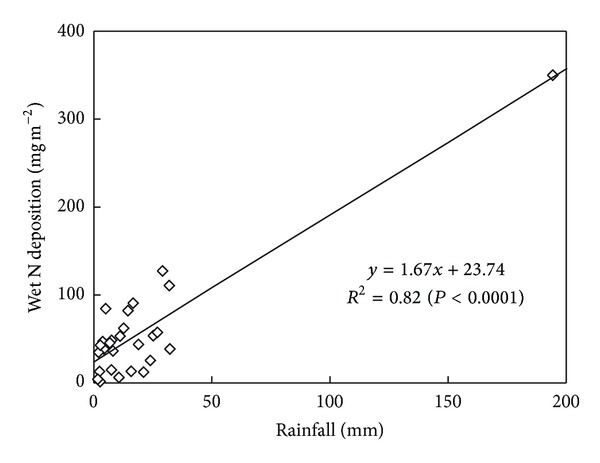
Relationship between precipitation and the content of nitrogen in wet deposition.

The dry and wet atmospheric nitrogen depositions were ~32% and ~68% of the total atmospheric nitrogen deposition, respectively (Table 1). The content of nitrogen in dry deposition was the highest in May when the wind was strong in spring in the YRD. With the precipitation increasing and wind becoming weak in summer, the proportion of wet nitrogen deposition increased (Figure 4). When the peak of precipitation occurred in August, the content of wet nitrogen deposition achieved the maximum value (675.64 mg m^−2^), of which the contribution reached 85.88%. With the precipitation decreasing dramatically from September, contribution of wet nitrogen deposition to the total atmospheric nitrogen deposition gradually declined (Figure 4). Further analysis revealed that there was significant positive relationship between nitrogen content in wet deposition and the precipitation (*R* ^2^ = 0.82) (Figure 5).

#### 3.1.3. Contribution of Atmospheric Deposition for Soil Nitrogen

The average contents of NO_3_^−^–N and NH_4_^+^–N in topsoil (0–10 cm) were 493.49 mg m^−2^ and 822.36 mg m^−2^, respectively (Table 2). The attribution rates of atmospheric NO_3_^−^–N and NH_4_^+^–N depositions ranged 3.73%–80.18% and 4.77%–77.47%, respectively. The maximum of attribution rates of atmospheric NO_3_^−^–N and NH_4_^+^–N deposition for that in topsoil both appeared in August (80.18% and 77.47%, resp.) (Table 2). The high attribution rates of atmospheric NO_3_^−^–N deposition for topsoil nitrogen reached 78.04% in May, while that of NH_4_^+^–N was the lowest (no more than 5%). On the contrary, the attribution rates of atmospheric NO_3_^−^–N deposition for topsoil nitrogen was the lowest in November (3.73%), while that of NH_4_^+^–N reached 20.83%. The average monthly attribution rates of atmospheric deposition of NO_3_^−^–N and NH_4_^+^–N for corresponding nitrogen in 0–10 cm soil layer in the plant growing season were about 31.38% and 20.50%, respectively (Table 2).

### 3.2. Discussions

The ionic composition of atmospheric depositions varied in different regions [12, 26, 31]; that is, Ca^2+^ and SO_4_^2−^ were the most abundant cation and anion in urban Beijing [32] and Northern Italy [31]. By contrast, the predominant cation and anion in wet and dry atmospheric depositions in the YRD were Na^+^ and SO_4_^2−^, respectively (Figure 2). It was closely related to that high salt content in fluvoaquic soil and saline soil which were widely distributed in the YRD [27, 33]. Our results showed that the most ratios of anions to cations in atmospheric deposition were less than 1, probably due to some anions such as F^−^, Br^−^ and short chain organic anions were not measured in this study [34, 35].

The atmospheric nitrogen deposition has been of great concern since 1980s, mainly due to acid rain and its negative effect on ecosystem [36–38]. Previous studies reported that the atmospheric nitrogen deposition only in growing season (2264.24 mg N m^−2^) was higher than total nitrogen deposition for the whole year [39]. To agree with that, a large amount of nitrogen deposition was received in coastal zone of the YRD from May to November (Table 1). The wet nitrogen deposition mainly occurred from June to August (Figure 4) because of precipitation (Figure 5). The wet atmospheric nitrogen deposition was more than 2 times of dry atmospheric nitrogen deposition in study region, which was similar with previous results in coastal zone of Barnegat Bay (>80%) [40].

Dentener and Crutzen [41] reported that anthropogenic emissions from domestic animals, fertilizer application, and biomass burning were thought to be the largest source of NH_4_^+^–N in atmospheric deposition. The monthly variations of atmospheric NH_4_^+^–N deposition results showed that both dry and wet atmospheric NH_4_^+^–N depositions were high in July and August (Figures 3(a) and 3(b)) when amount of fertilizer is applied for croplands. Another peak dry atmospheric NH_4_^+^–N deposition appeared in autumn (Figure 3(a)) probably because of the biomass burning in field. The dry atmospheric NO_3_^−^–N deposition was decreased from spring to autumn and the maximum values (113.89 mg m^−2^) appeared in May (Figure 3(a)). Its reason is probably that the NO_3_^−^–N of dry atmospheric deposition was strongly influenced by petrochemical industrial pollution which is transferred by wind from Dongying city. However the high NO_3_^−^–N of wet atmospheric deposition occurring in August (Figure 3(b)) was much related precipitation (Figure 5). The similar results were also reported in several studies monitored at similar latitude in China [22, 42]. The seasonal variation of NH_4_^+^/NO_3_^−^ ratio could reflect the deposited nitrogen source [43]. Compared with the developed region, the average NH_4_^+^/NO_3_^−^ ratio in atmospheric nitrogen deposition in this study (~1.16) was much less than that in Beijing area and Liaohe River Plain of Northeast China [22, 43] and similar to that in central New York [44] and Europe [11], suggested that the atmospheric nitrogen deposition in this region was affected by both agricultural activities and industrial activities.

Atmospheric nitrogen deposition has become a large source of nitrogen for terrestrial and aquatic ecosystems worldwide [7]. The total amount of nitrogen deposition and other environment-derived nitrogen in China was up to 18 Tg N year^−1^, equal to approximately 60% of the national nitrogen fertilizer consumption [9]. Atmospheric inputs of bioavailable nitrogen represented an imbalanced contribution to the new production of 8–20% in the Mediterranean coast of Israel [45]. The average monthly attribution rates of atmospheric deposition of NO_3_^−^–N and NH_4_^+^–N were about 31.38% and 20.50% for the contents of NO_3_^−^–N and NH_4_^+^–N in 0–10 cm soil layer, respectively (Table 2), suggesting that the atmospheric nitrogen deposition was one of the main sources of soil nitrogen in coastal wetland ecosystem in the YRD.

## 4. Conclusions

The cation of Na^+^ and anion of SO_4_^2−^ were major ionic compositions in dry and wet atmospheric deposition in the YRD. There were the significant relations of the total number of anions and the total number of cations in both dry atmospheric depositions (*P* < 0.005) and wet atmospheric depositions (*P* < 0.0001), respectively. The main form of atmospheric nitrogen input was wet deposition which accounted for 67.98% of the total atmospheric nitrogen deposition. Both dry and wet atmospheric NH_4_^+^–N depositions were high in July and August. The NO_3_^−^–N of dry atmospheric deposition was decreased from spring to autumn. There was a significant positive relationship between wet atmospheric nitrogen deposition and precipitation. The average NH_4_^+^/NO_3_^−^ ratio in atmospheric nitrogen deposition indicated that the atmospheric nitrogen deposition in this region was affected by both agricultural activities and industrial activities. Our results suggested that the atmospheric nitrogen deposition was one of the main sources of soil nitrogen in coastal wetland ecosystem in the YRD.

## Figures and Tables

**Table 1:** The monthly variation of atmospheric nitrogen deposition.

Month	Dry deposition		Wet deposition		NO_3_^−^–N		NH_4_^+^–N		TIN (mg m^−2^)
	N content (mg m^−2^)	% of TIN	N content (mg m^−2^)	% of TIN	Content (mg m^−2^)	% of TIN	Content (mg m^−2^)	% of TIN	
May	139.99	53.68	120.79	46.32	178.55	68.47	82.23	31.53	260.78
Jun.	103.62	27.39	274.77	72.61	242.70	64.14	135.69	35.86	378.39
Jul.	119.84	30.04	279.03	69.96	175.42	43.98	223.44	56.02	398.87
Aug.	111.12	14.12	675.64	85.88	280.22	35.62	506.54	64.38	786.76
Sep.	77.70	41.99	107.34	58.01	97.72	52.81	87.32	47.19	185.03
Oct.	91.66	60.89	58.88	39.11	77.15	51.25	73.38	48.75	150.54
Nov.	81.00	77.98	22.88	22.02	32.37	31.17	71.50	68.83	103.87

Total	724.92	32.02	1539.32	67.98	1084.13	47.88	1180.11	52.12	2264.24

**Table 2:** Atmospheric N deposition contributes to N inputs to local soil.

Month	Topsoil		Atmospheric deposition	
	NO_3_^−^–N (mg m^−2^)	NO_4_^+^–N (mg m^−2^)	% of NO_3_^−^–N content in topsoil	% of NO_4_^+^–N content in topsoil
May	228.78	1722.77	78.04	4.77
Jun.	706.59	976.09	34.35	13.90
Jul.	786.59	602.23	22.30	37.10
Aug.	349.47	653.89	80.18	77.47
Sep.	281.27	816.33	34.74	10.70
Oct.	234.77	641.91	32.86	11.43
Nov.	866.97	343.27	3.73	20.83

Average	493.49	822.36	31.38	20.50
